# Use of an Insulation Layer on the Connection Tracks of a Biosensor with Coplanar Electrodes to Increase the Normalized Impedance Variation

**DOI:** 10.3390/bios9030108

**Published:** 2019-09-16

**Authors:** Arthur Luiz Alves de Araujo, Julien Claudel, Djilali Kourtiche, Mustapha Nadi

**Affiliations:** Institut Jean Lamour, Lorraine University (CNRS—UMR 7198), 54011 Nancy, France; alvesdea1@univ-lorraine.fr (A.L.A.d.A.); julien.claudel@univ-lorraine.fr (J.C.); djilali.kourtiche@univ-lorraine.fr (D.K.)

**Keywords:** biosensor, impedance spectroscopy, coplanar electrodes, biological cell detection

## Abstract

New technologies, such as biosensors and lab-on-a-chip, are reducing time consumption and costs for the detection and characterization of biological cells. One challenge is to detect and characterize cells and bacteria one by one or at a very low concentration. In this case, measurements have very low variations that can be difficult to detect. In this article, the use of an insulation layer on the connection tracks of a biosensor with coplanar electrodes is proposed to improve a biosensor previously developed. The impedance spectroscopy technique was used to analyze the influence of the insulation layer on the cutoff frequencies and on the normalized impedance variation. This solution does not induce changes in the cutoff frequencies, though it permits improving the normalized impedance variations, compared to the same biosensor without the insulation layer.

## 1. Introduction

Characterizing biological cells or tissues by their biophysical properties is an important way to identify pathologies and to improve disease treatment or to detect pathogens [1]. Knowledge of electromagnetic properties of cells can, for example, provide early signals of disease or abnormal conditions in the human body, like blood diagnosis [2]. However, this characterization is usually done in a laboratory using expensive equipment and lengthy procedures. Reducing these constraints is one of the main interests of current research. In particular, one can mention recent works focused on the fast detection of very low bacteria concentration like *Escherichia coli* [3]. New technologies that have been developed to characterize cells faster, cheaper, and more accurately include electrorotation [4], impedance flow cytometry [5,6,7], electrical bio-impedance spectroscopy (BIS) [8,9,10], and electrochemical immunosensors [11]. Thus, these technologies are valuable potential markers for identifying cancers [12,13], bacteria [14] or parasites (as malaria) [15], toxins [16], the nature of tissues [17], and DNA properties [18].

In this way, the BIS technique with single biological particles suspended in physiological media [19], with the use of magnetic nanobeads and screen-printed interdigitated electrodes [5,20], or with microwell arrays between transparent conducting electrodes within a microfluidic channel to deliver and extract cells [21], is able to detect and characterize cells and bacteria. Combined with microfluidics devices, impedance spectroscopy can provide a powerful tool for sorting, analyzing, counting, or discriminating cells [5,22,23].

The limit of the detection is the lowest number of biological cells that can be measured by a biosensor. One way to improve it is to reduce the electrode size. Thus, the rate of detection is also reduced. As presented in a previous work [14], using an electrode array increases the rate of detection; however, the electrode connection tracks (principally for the electrodes in the center of the array) create a current leakage to the electrolyte or the substrate, which reduces the normalized impedance variation. Current methods to reduce the electrical current leakage are growing a layer of SiO_2_ onto the substrate (this method is used when the substrate is silicon) and using a passivation layer with polymer compounds, alternating Si_3_N_4_ and SiO_2_ or using an individual Si_3_N_4_ layer [24,25].

The current leakage effects were already significantly lowered by factor 2, with the reduction of the connection track surface exposed to the electrolyte as shown in [26].

To further reduce the current leakage effects, we propose in this paper, in addition to the previous optimization [26], the use of an insulation layer on the connection track, and we analyze its influence on the cutoff frequencies and on the normalized impedance variation. To analyze this, an electrical model was developed. Simulations using the Finite Element Method (FEM) were done. To confirm the theoretical and simulation analysis, experimental results are presented and analyzed. Two designs of four coplanar electrodes array each were designed: The first one without the insulation layer on the connection track, and the second one with a SiO_2_ insulation layer on it.

The biosensor includes a microchannel to center the sample on the electrodes, and the measurements were taken in static mode (the cell being immobilized) for a spectroscopic analysis. To place the sample on the electrode and to complete the static measurements, a low-cost system based on Pascal’s law was used.

The second section of this work presents the Materials and Methods in which the principle of the biosensor, the electrical model, the analytical and simulation analysis, the fabrication procedures, the samples that were used, and the schematic of the experimental results are shown. In the third section, the analytical and simulation results as well as the experimental results are described. The fourth section concludes by covering the influence of using an insulation layer on the cutoff frequencies and on the normalized impedance variation.

## 2. Theoretical Aspect

### 2.1. Electrodes Structure

**Figure 1:**
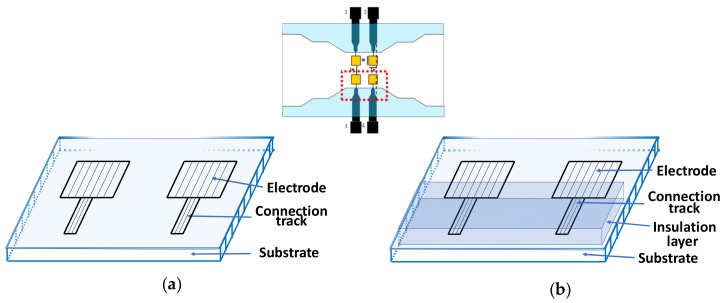
Electrode structures (**a**) without and (**b**) with an insulation layer on the electrode connection track.

The structure of the sensor is given in Figure 1. It is composed of an array of 4 coplanar microelectrodes placed into a microchannel. As already discussed in our previous work [26], the effects of current leakage are more prevalent for a couple of electrodes placed on the same side. That is why our investigation was focused on this pair of electrodes. The previous biosensor (BS1) (biosensor without an insulation layer on the connection tracks) and the new one (BS2) (biosensor with an insulation layer on the connection tracks) electrode structures are given in Figure 1a,b. The insulation layer is proposed to reduce the parasitic effects of the electrode connection track on the normalized impedance variation.

### 2.2. Electrical Model

**Figure 2:**
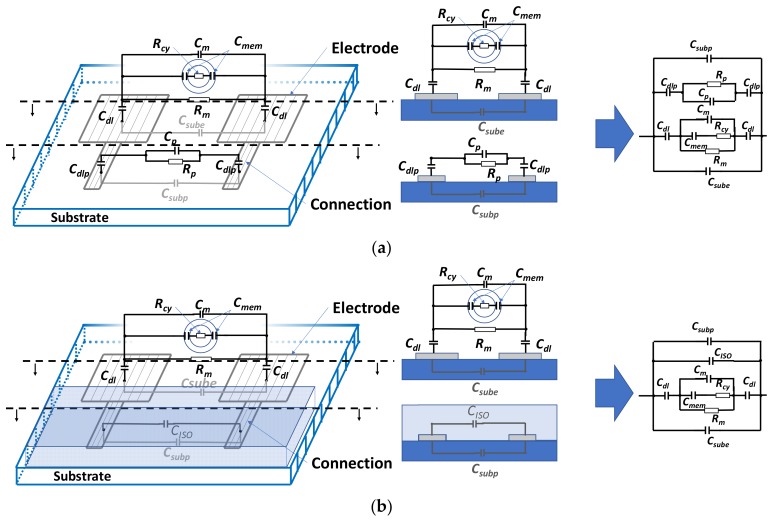
Schematic and electrical model of a biological cell between two identical coplanar electrodes for a biosensor (**a**) without the insulation layer and (**b**) with an insulation layer on the connection tracks.

The electrical model for two identical coplanar electrodes is shown in Figure 2a. Two identical coplanar electrodes with an insulation layer on the electrode connection tracks are shown in Figure 2b. The electrical components of the model are described in Table 1. The medium and the biological cell electrical components *R_m_*, *R_cy_*, *C_mem_*, and *C_m_* could be expressed in terms of the sample intrinsic parameters [19], as given by Equations (1)–(4). The intrinsic parameters of the electrical components are described in Table 2. Those are the parameters of interest that we need to extract from impedance measurement.

*C_dl_* is the metal–medium capacitance interface. *C_sub_* and *C_subp_* represent the capacitive effects of the substrate (under electrodes and tracks), independently of the use of the insulation layer. *R_p_*, *C_dlp_*, and *C_p_* represent the electrical contribution of the medium under investigation and the metal–medium interface on the connection track for BS1 (Figure 2a). When the insulation layer is used (BS2), the electrical effect is modeled by *C_ISO_* (Figure 2b). All these effects depend on the substrate permittivity and conductivity, and other used materials, as well as the electrode’s shape factor and the connection track, as described in Appendix A.

All these effects could influence the measured impedance and can be considered as undesired or parasitic effects. For example, parasitic capacitive effects can act as an electronic filter and decrease the measured frequency band until short-circuiting the impedance of the sample.

(1) $$R_{m}=\frac{1}{\sigma_{m}\left(1-\frac{3\emptyset }{2}\right)K\;};$$

(2) $$C_{m}=\epsilon_{m}\left(1-\frac{3\emptyset }{2}\right)K;$$

(3) $$R_{cy}=\frac{4\left(\frac{1}{2\sigma_{m}}+\frac{1}{\sigma_{cyt}}\right)}{9\emptyset K};$$

(4) $$C_{mem}=\frac{9\emptyset rC_{mem,S}}{4}K.$$

### 2.3. Cutoff Frequencies

**Figure 3:**
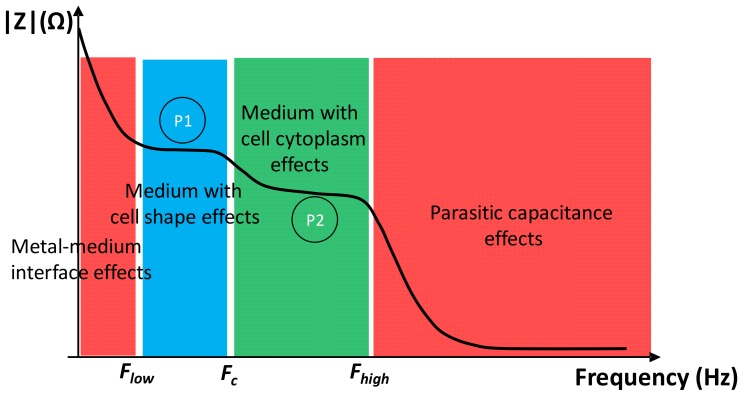
Schematic drawing of the impedance versus the frequency bands. The red zones represent the undesired intrinsic effects, and the blue and green zones represent the effects of the medium and of the biological cell.

The standard impedance spectrum of a micro-sample has four regions. In each region, there is a preponderant effect. The different zones are shown in Figure 3. At the lower frequencies (up to several tens of kHz), the effects of a double layer (metal–medium interface effect) are preponderant. These effects depend on electrode surfaces and occur at higher frequencies in the case of measurements performed on a microscopic scale. At medium frequency (several tens of kHz to several MHz), represented by blue and green regions, the impedance depends on the medium and cell/particle. The first plateau (P1) depends on cell size, and the second (P2) on cell cytoplasm properties. In frequencies above several MHz, the parasitic effects due to the substrate and electrode connections are preponderant. 

The transition frequencies between each region could be calculated as cutoff frequencies by analogy with discrete circuits. The transition between the effect of the double-layer capacitance and the medium plus cell effects occurs at *F_low_* frequency. The double-layer capacitances are in series with the impedance of the sample under test, and act as a high-pass filter, limiting the low-frequency band. The transition between the medium with cell shape effects and the medium with cell cytoplasm effects occurs at the frequency *F_c_*, generally around MHz for mammalian cells [27]. It is due to the *R_cy_*/*C_mem_* couple. Moreover, the transition between the sample effects and parasitic capacitances occurs at *F_high_* frequency. These capacitances act as a low-pass filter and limit the higher frequency band. According to our models, these cutoff frequencies can be expressed following Equations (5)–(9). They depend on the electrode material and on the design, except for *F_c_*, which depends only on the sample properties.

(5) $$F_{c}=\frac{1}{2\pi R_{cy}C_{mem}}.$$

(6) $$F_{low,BS1}=\frac{1}{2\pi \sqrt{C_{dl}R_{m}C_{dlp}R_{p}}}.$$

(7) $$F_{low,BS2}=\frac{1}{2\pi C_{dl}R_{m}}.$$

(8) $$F_{high,BS1}=\frac{R_{cy}R_{p}+R_{m}R_{p}+R_{m}R_{cy}}{2\pi R_{m}R_{cy}R_{p}\left(C_{sub}+C_{m}+C_{subp}+C_{p}\right)}.$$

(9) $$F_{high,BS2}=\frac{R_{m}+R_{cy}}{2\pi R_{m}R_{cy}\left(C_{sub}+C_{m}+C_{subp}+C_{iso}\right)}.$$

### 2.4. Normalized Impedance Variation

The normalized impedance variation ($\Delta {\left|Z\right|}_{n}$) represents the ratio of the impedance when a cell or a particle is present between the electrodes (*Z_cell_*) and the impedance without cell (*Z_ref_*), as described in Equation (10). The higher this parameter, the easier the detection of a biological cell.

(10) $$\Delta {\left|Z\right|}_{n}=\frac{Z_{cell}-Z_{ref}}{Z_{ref}}.$$

*Z_ref_* was chosen at the specific frequency *F_n_*, corresponding to the frequency where the resistive effect of the medium is predominant (*R_m0_*). This frequency can be calculated using the analytical model or extracted from the spectrum measurement (corresponding to a local extremum in phase). 

Therefore, $\Delta {\left|Z\right|}_{n}$ for the sensors BS1 and BS2 can be expressed using Equations (11) and (12) when measurements are performed in the first plateau *P1* or using Equations (13) and (14) when measurements are performed on the second plateau *P2*. For non-conductive particles, such as microbeads, only Equations (11) and (12) are needed since *σ_cy_* can be neglected.

(11) $$\Delta {\left|Z\right|}_{n,BS1,P1}=\frac{(R_{m0}+R_{p})R_{m}}{(R_{m}+R_{p})R_{m0}}-1.$$

(12) $$\Delta {\left|Z\right|}_{n,BS2,P1}=\frac{R_{m0}R_{m}R_{cy}R_{p}+R_{m}R_{cy}R_{p}{}^{2}}{R_{m0}R_{cy}R_{p}{}^{2}+R_{m0}R_{m}R_{p}{}^{2}+R_{m0}R_{m}R_{cy}R_{p}}-1.$$

(13) $$\Delta {\left|Z\right|}_{n,BS1,P2}=\frac{R_{m}}{R_{m0}}-1.$$

(14) $$\Delta {\left|Z\right|}_{n,BS2,P2}=\frac{R_{m}R_{cy}}{(R_{m}+R_{cy})R_{m0}}-1.$$

## 3. Simulations

### 3.1. Simulations Setup

To analyze the effects of the insulation layer on the cutoff frequencies and on the impedance variations, both analytical and FEM simulations were performed. The simulations were focused on the sensing area, composed of a pair of coplanar microelectrodes in a microchannel (60 µm in width, and 20 µm height). The dimensions of the electrodes are 10 × 10 µm², spaced by 10 µm, and the connection track dimensions exposed to the electrolyte are fixed lengths of 5 µm, 10 µm, and 20 µm. An insulation layer with a thickness of 150 nm was computed for the new generation sensor. Materials and cell properties are given in Table 3. Cell electrical properties correspond to general parameters of living cells [27].

FEM simulations were performed using COMSOL (COMSOL Multiphysics^®^ COMSOL AB, Stockholm, Sweden). More details about the FEM simulation model are given in Appendix B.

Analytical simulations were performed by computing Equations (1)–(4) and Equations (A1)–(A7) with MatLab (Mathworks, Natick, MA, USA), and using the shape factor of electrodes/connection tracks obtained with FEM simulations.

### 3.2. Simulation Results

**Figure 4:**
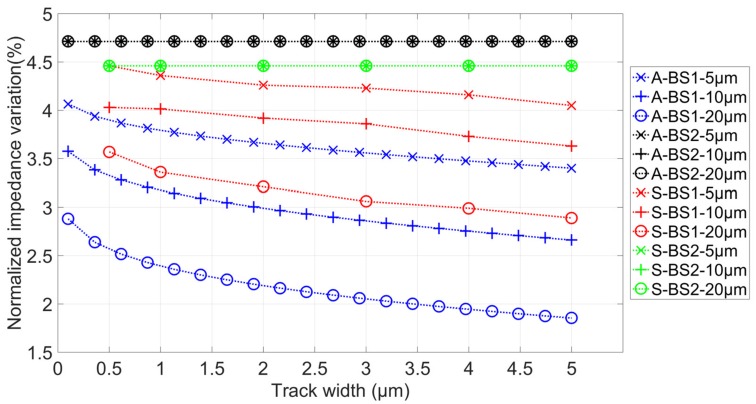
Normalized impedance variation as a function of track width for three different connection length (5 µm, 10 µm, and 20µm), with and without an insulated layer. “A” corresponds to analytical calculation, and “S” to FEM simulation results. Green and black curves are superimposed.

The first simulation was performed at 10 kHz with an 8 µm diameter cell. At this frequency, the cell can be considered as a non-conductive particle, and only its size influences impedance variations. Results are given in Figure 4. Both analytical and FEM simulations follow the same trend and are in good accordance. It clearly appears that impedance variations are not influenced by connection tracks geometries when the insulated layer is present. Furthermore, impedance variations can decrease with a factor higher than 2 with the increase of track width. These differences are due to current leakages that occur between tracks. The differences between analytical and simulated results can be explained by the linearization of the analytical model and the possible approximations of FEM (limit conditions). The analytical model does not take into account the possible electric field distortions.

**Figure 5:**
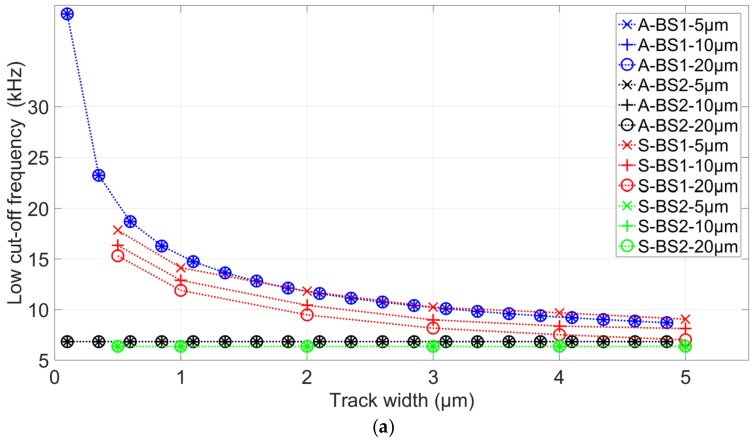

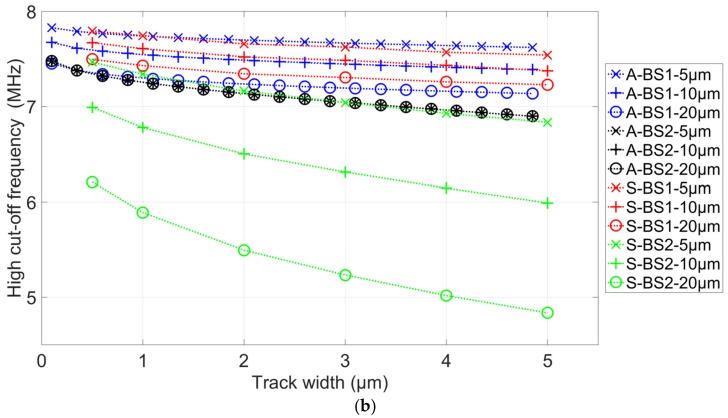
Simulated (**a**) low-cutoff frequency and (**b**) high-cutoff frequency as a function of track width for three different connection length (5 µm, 10 µm, and 20µm), with and without an insulated layer. “A” corresponds to analytical calculation, and “S” to FEM simulation results.

Second simulations were performed with an 8 µm cell to determine the impact of the insulating layer on the low- and high-cutoff frequencies (Figure 5). Both analytical and FEM simulations follow the same trend and are in good accordance. Like the previous simulation, *F_low_* is not influenced by connection tracks geometries when the insulated layer is present. Furthermore, *F_low_* can increase by up to more than three times with the decreasing of track width. This is caused by the couple (R_p_:C_dl_), which is present only without an insulated layer. This increase of *F_low_* can reduce the frequency band of interest. 

Even if both impedance variation and low-cutoff frequency are better with tracks insulation, the high-cutoff frequency is lower with this solution. However, this decreasing of high cutoff stays low compared to the other criteria and can be optimized using the smallest tracks. It appears that the best optimization solution is to insulate tracks and to reduce their widths as much as possible. 

## 4. Material and Methods

### 4.1. Sensors Fabrication

**Figure 6:**
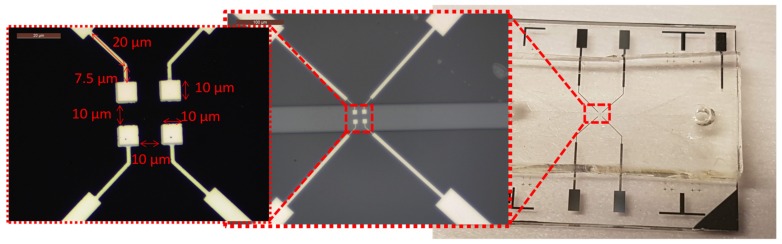
Image of the built biosensor with electrodes and connection track dimensions. From right to left: Complete biosensor with electrical pads and microfluidic-macrofluidic interface; Microfluidic channel and electrodes; Electrodes and connection track dimensions.

The biosensors were fabricated using a standard photolithography process, as already described in Supplementary file, “Sensors fabrication.” The biosensors fabrication is divided into two parts: The first one is the functional part with platinum electrodes structuration on a glass substrate, and the second one is a microfluidic channel molded in polydimethylsiloxane (PDMS). The bonding of these two parts is done by a surface treatment of the PDMS with Corona plasma. For the new biosensor, a standard lift off the deposition of SiO_2_ was added to insulate electrode tracks. Figure 6 shows the image of the built biosensor with electrodes and connection track dimensions.

### 4.2. Samples Preparation

Microbeads of 10 μm in diameter (Polybead^®^ Black Dyed Microspheres, Polysciences Europe GmbH, Germany) were used as reference particles for our measurements. The microbeads were diluted in dechlorinated tap water (proportion of 1:10) to establish a laboratory situation that was closer to an onsite measurement situation. Tap water is representative of general drinking water in terms of mineral composition and conductivity. With the use of diluted standard buffer solution, only conductivity can be respected. The tap water was electrically characterized in our laboratory using high-precision liquid probe 16452A Liquid Test Fixture (Keysight Technologies^®^, Santa Rosa, CA, USA) and was found to have an electrical conductivity of 300 μS/cm@100 kHz@24 °C.

### 4.3. Experimental Setup

We used the same experimental setup as the one given in [26] (Section 5.2). The measurement setup is composed of a micropositioner, a microscope with a CMOS camera, a Keysight E4990A impedance analyzer (Keysight Technologies, Santa Rosa, CA, USA), and a computer. The impedance analyzer uses the method of Auto-Balancing Bridge in which the electrodes are excited with a variable frequency electrical signal, and the impedance response is measured. This method is able to performe accurate measurements in a wide band of impedances and frequencies [28]. More details about the measurement setup are detailed in Appendix C.

### 4.4. Experimental Results

**Figure 7:**
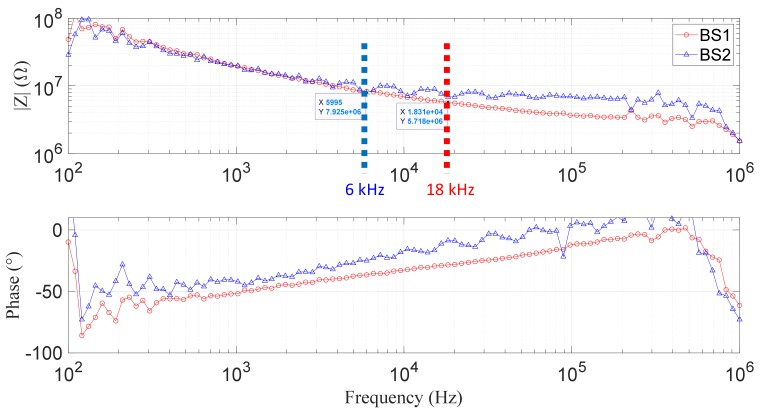
The impedance spectrum of the biosensor without the insulation layer B_s_ (**blue**) and with the insulation layer B_W_insu_ (**orange**).

First impedance measurements were done using the impedance analyzer in the bandwidth 100 Hz to 1 MHz, as shown in Figure 7. The *F_low_* (frequency in which the impedance fall stopped giving place to the first plateau) is around 6 kHz for the biosensor BS2 and around 18 kHz for the biosensor BS1. As predicted by the analytical and simulation analysis, the *F_low_* for the biosensor BS2 is lower than the *F_low_* for the biosensor BS1. It was not possible to obtain the *F_high_* due to the limitations of the measuring frequency band of the experimental setup, as can be seen in the impedance curve; from 200 kHz, the noise is very large. Therefore, we can say that *F_high_* is at least 200 kHz.

**Figure 8:**
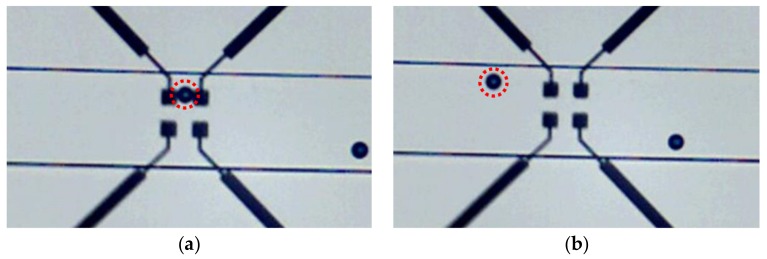
Microscopic image of the biosensor center (**a**) with a microbead between the electrodes for the Z_cell_ measurement and (**b**) without the microbead between the electrodes for the Z_ref_ measurement.

To obtain the normalized impedance variation we used the technique previously described in [26]. In this technique, microbeads are moved along the channel using a small pressure gradient and stopped between the electrodes, as it is shown in Figure 8a. *Z_cell_* impedance is measured when the cell is centered between the electrodes. The second time, the microbead is moved out, as shown in Figure 8b, and the reference impedance *Z_ref_* is measured.

**Figure 9:**
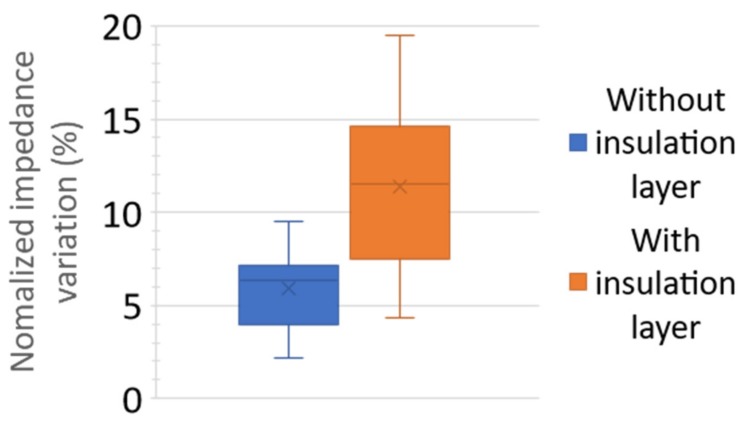
Statistical distribution of the experimental normalized impedance variation for the biosensors, BS1 and BS2.

The calculation of the normalized impedance variation for BS2 and BS1 was made at 60 kHz and 100 kHz, respectively, corresponding to the frequency where the resistive effect of the medium is predominant. These frequencies seem to be the more suitable because parasitic effects are minimal, and impedance depends principally on the sample under test. Results are shown in Figure 9 for the biosensors, BS1 and BS2. To simplify the lecture of the data, we plotted the statistical box with the normalized impedance variation. We completed 42 measurements: 23 with BS1 and 19 with BS2.

The normalized impedance variation is higher for the biosensor BS2 than for the biosensor BS1, as predicted by the analytical and FEM simulations. The center of the distribution of the biosensor BS2 is around 11.5% and for the biosensor BS1 around 6%, this means an improvement of up to 92% of the normalized impedance variation when an insulation layer is used on the connection tracks. The change in the center of distribution to the smaller value for BS1 is 65% and for BS2 is 62%. Compared to the larger value, the variation is 60% for BS1 and 70% for BS2. These similar variations on BS1 and BS2 occur because we do not control the placement of microbeads perpendicular to the electrodes. 

## 5. Discussion and Conclusions

To reduce the undesired effects of biosensor connection, we investigated in this paper the use of adding an insulation layer on the connection tracks. To analyze the effects of the use of the insulation layer on the normalized impedance variation and on the cutoff frequencies, an electrical model was developed, and simulations using FEM were performed. The analytical and simulation results were confirmed by experimental results.

When the dimensions of the electrodes are reduced, sensors are more suitable to detect/characterize a low quantity of biological cells, or even a single cell if electrodes have the same size order. Results obtained prove this ability to detect only the particle. However, the decreasing of electrode sizes and the increasing of electrode numbers occurred at the same time as increasing sensitivity to parasitic capacitance. The undesired connection effects can widely decrease the normalized impedance variation if the design is not optimized.

From the results, we conclude that the use of insulation layer on the connection tracks improves the normalized impedance variation. This study allowed us to confirm that the use of an insulation layer on the connection tracks can improve up to 92% the normalized impedance variation, compared to the biosensor without the insulation layer. The low-cutoff frequencies can decrease significantly when an insulation layer is used, thus increasing the frequency band of interest. However, the high-cutoff frequency decreases too, but this decrease stays moderate and does not disturb the measurement of the plateau. 

In the case where the number of electrodes in the array is higher than 3 × 3, the electrodes closer to the center of the array will have more connection tracks exposed to the electrolyte and will have a lower normalized impedance variation. To avoid this reduction of normalized impedance variation, the use of the insulation layer on the connection tracks is a solution to avoid it.

## Figures and Tables

**Table 1:** Description of the electrical model components.

Component	Description	Unit
*C_dl_*	Capacitance of the double-layer effect between the electrode and the medium	F
*C_m_*	Medium capacity	F
*R_m_*	Medium resistance	Ω
*R_cy_*	Cymiddlelasmic cell resistance	Ω
*C_mem_*	Membrane cell capacity	F
*C_sub_*	Capacitance of substrate under electrode	F
*C_subp_*	Capacitance of substrate under connection tracks	F
*R_p_*	Parasitic resistance of the connection track for the biosensor BS1	Ω
*C_dlp_*	Double-layer capacitance of the connection track for the biosensor BS1	F
*C_p_*	Parasitic capacitance of the connection track for the biosensor BS1	F
*C_ISO_*	Insulation capacitance of the connection track for the biosensor BS2	F

**Table 2:** Intrinsic parameters of the electrical components.

Component	Description	Constant Values	Unit
*Φ*	Volume fraction (ratio between the cell volume/measurement volume)	0.03	-
*K*	Shape factor	8.38	µm
*σ_m_*	Medium conductivity	1	S/m
*σ_cyt_*	Cell cymiddlelasm conductivity	1	S/m
*ε_m_*	Medium permittivity	78 × 8.85 × 10^−12^	F/m
*r*	Cell radius	From 3 to 9	µm
*C_mem,s_*	Cell membrane surface capacitance	1	µF/cm^2^
*K_p_*	Connection track shape factor	From 2.3 to 22.5	µm
*ε_sub_*	Substrate permittivity	4.2 × 8.85 × 10^−12^	F/m
*C_0_*	Superficial double-layer capacitance	0.01	F/m^2^
*A_ele_*	Electrode area	100	µm²
*A_p_*	Connection track area	From 0.5 to 100	µm²

**Table 3:** Electrical parameters used for Finite Element Method (FEM) simulations.

Material	Conductivity [S/m]	Relative Permittivity
Glass substrate	10^−13^	4.2
Medium (tap water)	0.03	78
Insulation (Si02)	10^−13^	4.1
Cell cymiddlelasm	1	78
Cell membrane	10^−13^	847 with 750 nm thickness (fixed to obtain 1 µF/cm²)
Polystyrene beads	10^−13^	2.4

**Figure A1:**
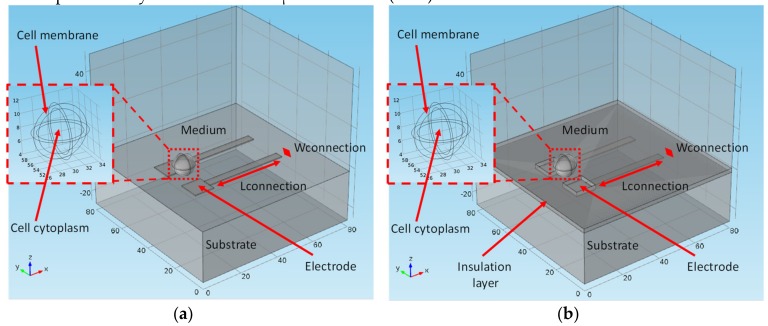
Simulation blocks and description of the components for the biosensor (**a**) without the insulation layer on the connection and (**b**) with the insulation layer on the connection tracks.

**Figure A2:**
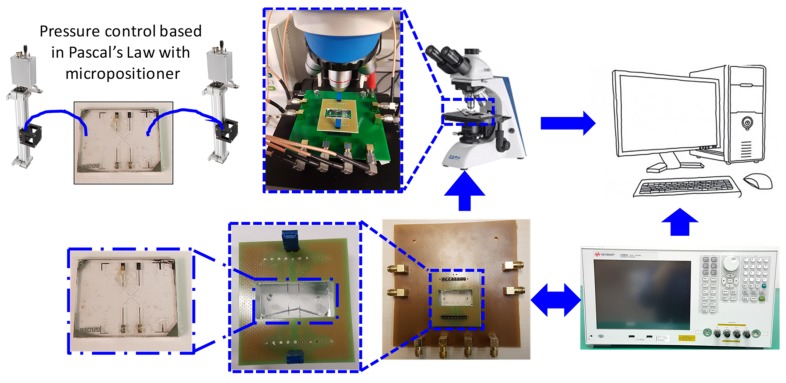
Schematic of the measurement setup composed of a micropositioner, a microscope with a CMOS camera, a Keysight E4990A impedance analyzer (Keysight Technologies, Santa Rosa, CA, USA), and a computer.

**Figure A3:**
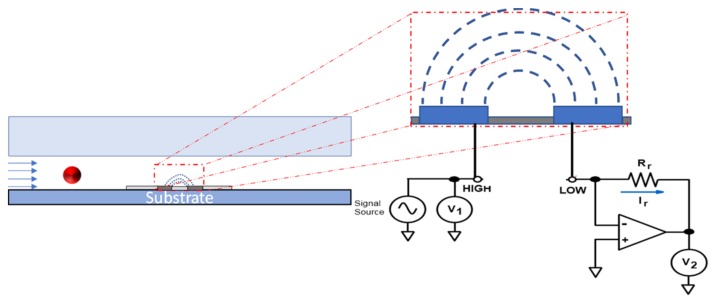
Measurement principle and method of coplanar microsensor. Microbeads are inserted into the microfluidic channel using syringe and needle, and the impedance measurements are performed using the method of Auto-Balancing Bridge.

## Supplementary Materials

The following are available online at https://www.mdpi.com/2079-6374/9/3/108/s1.

## Appendix A

The connection track electrical components *C_subp_*, *C_p_*, and *R_p_*; the metal–medium capacitance interfaces, *C_dl_*, and *C_dlp_;* the substrate capacitance under the electrodes *C_sub_*; and the insulation capacity C_ISO_ are described in Equations (A1) to (A7), respectively.

(A1) $$C_{subp}=\epsilon_{sub}K_{p}\;.$$

(A2) $$C_{p}=\epsilon_{m}K_{p}.$$

(A3) $$R_{p}=\frac{1}{\sigma_{m}K_{p}}.$$

(A4) $$C_{dl}=C_{0}A_{ele}\;.$$

(A5) $$C_{dlp}=C_{0}A_{p}\;.$$

(A6) $$C_{sub}=\epsilon_{m}K\;.$$

(A7) $$C_{iso}=\epsilon_{iso}K_{p}\;.$$

## Appendix B

In our simulations, cytoplasm is considered an ionic solution, mainly composed of minerals and other substances dissolved in water. It can be modeled as a resistive material with a fixed conductivity. Cell membrane is generally considered an insulate material. Due to its very small thickness, around 10 nm, it can be modeled by a surface capacitance (around 1 µF/cm²) [5]. We choose to model the cell using two concentric spheres to form the cytoplasm and cell membrane, as shown in Figure A1 [19,26]. Due to the high size differences between membrane thickness and global cell size, we decided to increase membrane thickness to 0.75 µm to reduce model complexity and computational costs. To keep the same surface capacitance (1µF/cm²), the membrane relative permittivity is adapted to 847 [26].

The COMSOL simulation blocs for the biosensor B_s_ is shown in Figure A1a and for the biosensor B_W_insu_ is shown in Figure A1b. The thickness used for the simulation of the insulation layer is 0.5 µm and has the same surface capacity as the experimental one. To do it, the electrical permittivity of the insulation layer was increased to 16 to obtain the same surface capacitance as the real one with a relative permittivity of 4.12 for a 0.15 µm thickness (SiO_2_).

## Appendix C

Figure A2 shows a schematic of the measurement setup. It is centered on a precision impedance analyzer Keysight E4990A (Keysight Technologies, Santa Rosa, CA, USA), driven by a computer. A pressure control system and a microscope with a CMOS camera are used for micro-positioning cell samples. The Liquid Test Fixture 16452A (Keysight Technologies, Santa Rosa, CA, USA) is used in accompaniment to first perform the characterization of tap water.

Figure A3 illustrates the measurement principle as well as the method for the impedance spectroscopy of a biosensor with coplanar microelectrodes. Microbeads are inserted into the microfluidic channel using a syringe and a needle. To centralize microbeads on microelectrodes, a low-cost system based on Pascal’s law with micropositioner is used to move microbeads in the micrometer scale. At the entrance and exit of the microfluidic channel, a capillary tubing is inserted, and at the other side of the capillary tubing, needles are inserted. We place the input and output needles at the same height for balance and move a needle up or down with the micropositioner to cause a small variation in potential, and thus, a small variation in pressure in the microchannel. This small variation in pressure is sufficient to move the sample in the microchannel. When the microbeads are placed between the electrodes, the impedance measurements are done.
