# Supplementary material for: Use of an Insulation Layer on the Connection Tracks of a Biosensor with Coplanar Electrodes to Increase the Normalized Impedance Variation
Source: Biosensors (Basel). 2019 Sep 16;9(3):108. doi: 10.3390/bios9030108 (PMC6784382; doi:10.3390/bios9030108)
Supplement: Supplementary file 1 [file biosensors-09-00108-s001.pdf]

biosensors

Detailed fabrication of biosensors

Arthur Luiz Alves de Araujo, Julien Claudel, Djilali Kourtiche and Mustapha Nadi *

Institut Jean Lamour, Lorraine University (CNRS—UMR 7198), 54011 Nancy, France;

Biosensors were fabricated using standard lithography process as already described in [26]. The biosensors fabrication is divided into two parts, the first one is the functional part with platinum electrodes structuration on a glass substrate and the second is a microfluidic channel molded in polydimethylsiloxane (PDMS). The bonding of these two parts is done by a surface treatment of the PDMS with Corona plasma.

1. Electrodes structuration

The realization process steps of the functional part is shown in Figure S1 and describe below. Steps 1 to 5 are common to both BS1 and BS2 biosensors. The tow lasts steps are using for BS2 insulating layer

1. The glass substrates are cleaned with detergent (RBS), acetone, and rinsed with isopropanol to remove all organic and inorganic materials.
2. To have a good adhesion of platinum to the substrate, tantalum was used as an adhesive layer. Both tantalum and platinum were deposit in all substrate surface using AC450 sputtering system to obtain 150nm thickness.
3. 1µm of S1813 photoresist was deposit on platinum surface by spin coating and electrodes was structured by UV insulation of S1813 resin with MJB4 mask aligner (SUSS MicroTec SE, Garching near Munich, Germany).
4. The unprotected metal surface was etching by ion beam process using 4WAVE IBE system (4Wave Inc., Sterling, VA, USA). The thicker S1813 layer able to resist longer time than platinum/tantalum layer during etching.
5. For the insulation deposition on the electrode connections, it was done by the lift-off process of SiO_2.
6. Negative pattern of insulated layer was structured using LOR3A photoresist and MJB4 mask aligner.
7. S_i0_2 insulated layer was deposit by sputtering (AC450) and lift-off.

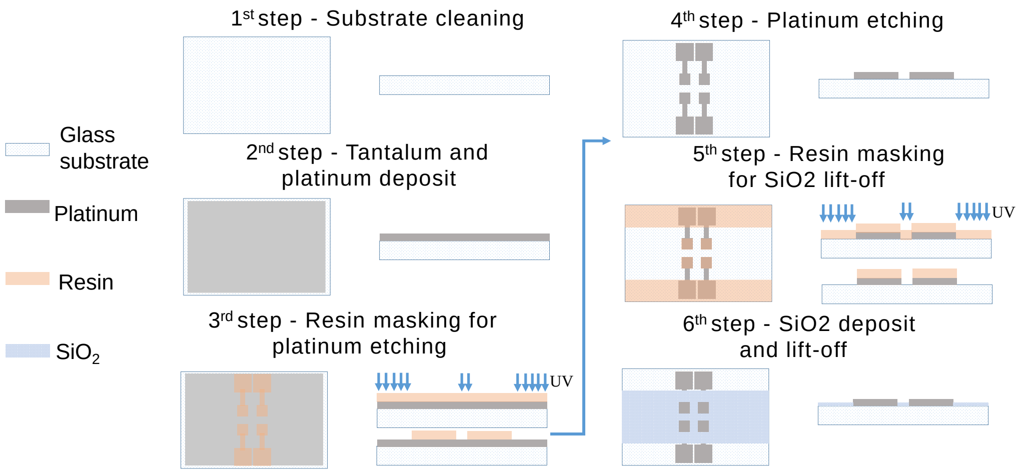

Figure S1 - Schematic of the realization process for the functional part.

2. Channel structuration

Figure A2 shows a schematic of the microfluidic channel realization. The microfluidic channel realization is made using the organomineral polydimethylsiloxane polymer (PDMS) and a mold.

The mold was made with the SU-8 photopositive thick resin on a silicon substrate following steps 1 to 3, described below:

1. The silicon substrate is cleaned with detergent (RBS), acetone, and rinsed with isopropanol to remove all organic and inorganic materials.
2. To have a good adhesion a primer was deposit by spin coating. SU-8 negative photoresist was also deposit by spin coating to obtain a thickness of 20µm.
3. Negative pattern of microchannel was structured by UV insulation of SU-8 resin with MJB4 mask aligner.

After mold realization, it is possible to structure any microchannel we want by molding following steps 4 and 5 :

4. Liquid PDMS was mix with curing agent (ratio 10:1), poured into the mold and placed into a vacuum chamber to remove bubbles.
5. After a curing time of 10 (at 150°C) to 48h (at 25°C) PDMS can be demolding.

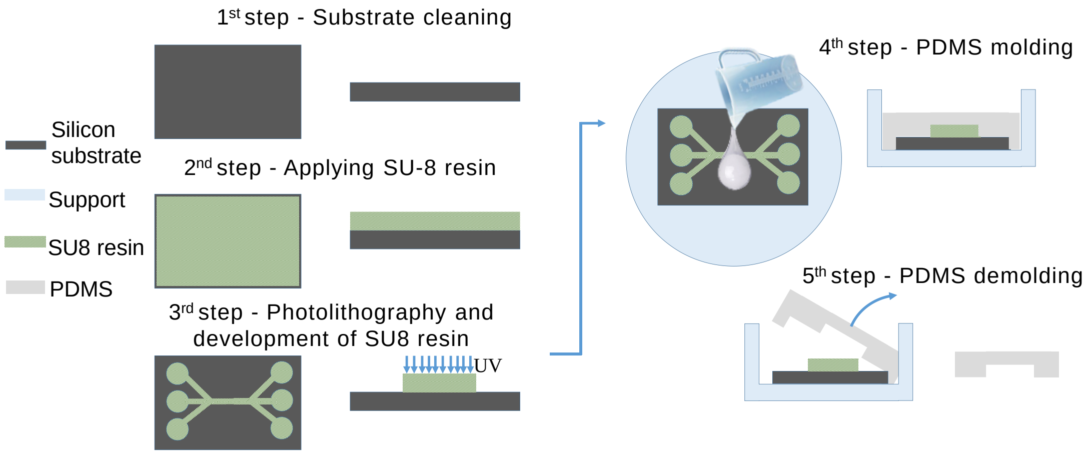

Figure A2 - Schematic of the realization process for the microfluidic part. The microfluidic channel is realized using (PDMS) and a mold.

3. Sensors assembly

The final step consist in chemically bond structured PDMS on glass substrate. This step was realized by functionalization of PDMS surface by plasma treatment. To do it, PDMS was cured with Corona plasma during 15 sec to modify it surface properties (PDMS became hydrophilic). Glass substrate was dried on hotplate and cleaned using O_2 plasma chamber. Both parts are assembly and cured on hotplate during 15 min at 115°C. The obtained sensor is presented in Figure S3.

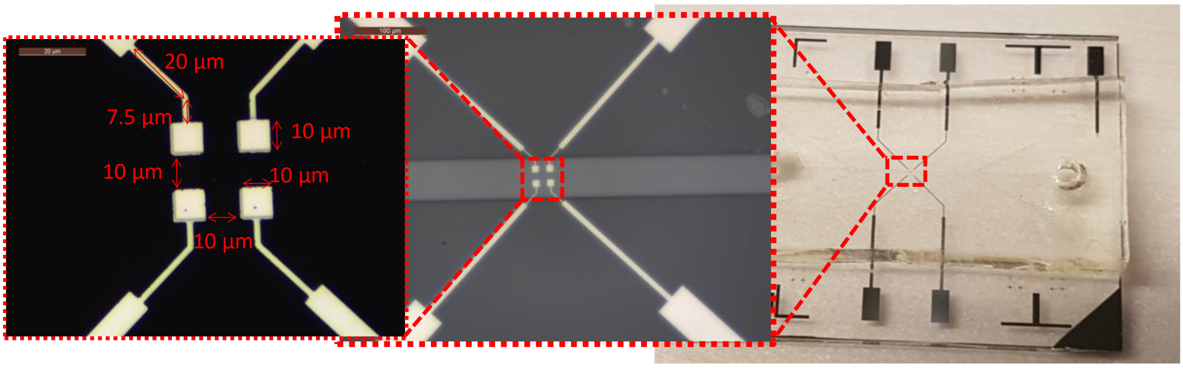

Figure S3 - Image of the realized biosensor with electrode and connection track dimensions. From right to left: Complete biosensor with electrical pads and microfluidic-macrofluidic interface; Microfluidic channel and electrodes; Electrodes and connection tracks dimensions.

2018 by the authors. Submitted for possible open access publication under the terms and conditions of the Creative Commons Attribution (CC BY) license (http://creativecommons.org/licenses/by/4.0/).
